# Suppression of zinc finger protein 467 alleviates osteoporosis through promoting differentiation of adipose derived stem cells to osteoblasts

**DOI:** 10.1186/1479-5876-10-11

**Published:** 2012-01-17

**Authors:** Li You, Ling Pan, Lin Chen, Jin-Yu Chen, Xiaoping Zhang, Zhongwei Lv, Da Fu

**Affiliations:** 1 Department of Osteoporosis, Shanghai First People's Hospital, Shanghai Jiao Tong University, 100 Haining Road, Shanghai 200080, China; 2 The Key Laboratory of Stem Cell Biology, Institute of Health Sciences, Shanghai Institutes for Biological Sciences, Chinese Academy of Sciences/Shanghai Jiao Tong University School of Medicine, 225 South Chongqing Road, Shanghai 200025, China; 3 Department of Nuclear Medicine, Shanghai 10th People's Hospital, Tongji University School of Medicine, 301 Middle Yanchang Road, Shanghai 200072, China

**Keywords:** Zfp467, ADSCs, osteoblast differentiation, osteoporosis, RNAi

## Abstract

Osteoblast and adipocyte are derived from common mesenchymal progenitor cells. The bone loss of osteoporosis is associated with altered progenitor differentiation from an osteoblastic to an adipocytic lineage. In this study, a comparative analysis of gene expression profiling using cDNA microarray and realtime-PCR indicated that Zinc finger protein 467 (Zfp467) involved in adipocyte and osteoblast differentiation of cultured adipose derived stem cells (ADSCs). Our results showed that RNA interference for Zfp467 in ADSCs inhibited adipocyte formation and stimulated osteoblast commitment. The mRNA levels of osteogenic and adipogenic markers in ADSCs were regulated by si-Zfp467. Zfp467 RNAi in ADSCs could restore bone function and structure in an ovariectomized (OVX)-induced osteoporotic mouse model. Thus Zfp467 play an important role in ADSCs differentiation to adipocyte and osteoblast. This has relevance to therapeutic interventions in osteoporosis, including si-Zfp467-based therapies currently available, and may be of relevance for the use of adipose-derived stem cells for tissue engineering.

## Introduction

Adult bone mass is maintained by an exquisite balance between bone formation by osteoblasts and bone resorption by osteoclasts [1]. Disruption of this delicate equilibrium can lead to osteoporosis (OP), a multifactorial, age-related metabolic bone disease characterized by reduction in bone mass, bone tissue microarchitectural deterioration, and increased fracture risk [2,3].

A variety of risk factors have been associated with osteoporosis, with emerging evidence suggesting a close association between bone aging, disease, and stem/progenitor cell defects [4,5]. Numerous animal and human studies have examined the links between mesenchymal or osteoprogenitor cell properties, aging, and osteopenia; however, many results to date have been contradictory and confusing.

Interpreting the results of these studies is further complicated by variations in the cell source site, isolation procedures, culture conditions, assay conditions, metrics, and developmental time-points being evaluated. The clearest trends are observed in murine osteoporosis/osteopenia models, including SAMP6 [6,7] and aged C57BL/6 [8] mice, which exhibit low bone mass and/or bone material and mechanical defects accompanied by altered mesenchymal progenitor properties (i.e., reduced numbers, proliferative capacity, or osteogenic differentiation capacity).

There are emerging evidences linking osteoporosis and stem cell defects, including osteoblast-progenitors (mesenchymal stem cells, MSCs) residing in the bone marrow [9], so it has been hypothesized that such cells from in vitro culture might be infused back to osteopenic subjects in order to replenish their stem cell pool, which would result in a positive bone balance and ultimately the regeneration of the osteopenic skeleton.

Mesenchymal stem cells, the precursor cells of adipocytes and osteoblasts [10], are found to play an important role in bone physiology and partly participate in the pathophysiology of osteoporosis. Indeed, in postmenopausal women who suffered from osteoporosis, MSCs were shown to exhibit a lower growth rate and have lower abilities to differentiate into osteogenic lineage than those from premenopausal women [11]. The increased volume of adipose tissue was also found in the bone marrow of postmenopausal women, implying the enhancement of MSCs' differentiation into adipocytes [12]. Additionally, because of increased cytokines from stromal cells and osteoblasts that regulated osteoclast generation due to estrogen loss, the number of osteoclasts increased and caused the elevated bone resorption [13].

Another emerging stem cell for treatment osteoporosis of is adipose-derived stem cells (ADSCs). The main benefits of ADSCs in therapeutic applications, as compared with bone marrow-derived MSCs, are that adipose tissue is readily accessible and relatively abundant, and the stem cell population can be easily harvested by simple methods, such as lipoaspiration or surgical resection, and can be rapidly expanded ex vivo [14]. ADSCs have also been shown to support differentiation of hematopoietic progenitors into myeloid and B lymphoid cells [15]. ADSCs-derived cellular therapy has been investigated with respect to a wide variety of human diseases, such as skeletal muscle disorders, cardiovascular disorders, and diabetes mellitus, and in bioengineering for tissue regeneration [16]. Additive support of ADSCs in tissue repair and regeneration has been reported to include differentiation into a proper cell lineage and paracrine mechanisms mediated by secreted cytokines and growth factors [17].

In this study, we characterize the initiation and adipocyte and osteoblast differentiation of cultured ADSCs at the cellular level. We hypothesized that Zinc finger protein 467 (Zfp467), a novel regulator of osteoblast and adipocyte commitment [18], would lead to adipocyte differentiation of ADSCs. We predicted that suppression of this factor by RNA interfere would mediate the expressions of specific osteogenic and adipogenic genes and alleviate ovariectomized (OVX)-induced osteoclasts formation and bone destruction.

## Materials and methods

### Isolation and culture of ADSCs

Mouse abdominal adipose tissues were obtained under approval from the Animal Research Guidelines of Shang Jiao Tong University School of Medicine. The lipoaspirate was incubated with collagenase type I solution (Worthington Biochemical, Lakewood, NJ) for 1 h at 37°C, and filtered through 250 μm filters. Following centrifugation, the stromal vascular fraction was resuspended in DMEM (HyClone, Logan, UT) supplemented with 10% FBS (HyClone), 100 U/ml penicillin, and 100 μg/ml streptomycin. ADSCs were cultured under a humidified atmosphere of 5% CO_2 _at 37°C for long-term culture in vitro and were capable of differentiating into adipocytes and osteogenic cells under specific induction [19].

Differentiation in cultured ADSCs was monitored including oil red O staining of lipid droplets in terminal adipocyte differentiation and von Kossa staining of calcium deposition in the extracellular matrix in terminal osteoblast differentiation.

### Vector construction

The lentiviral vector encoding Zfp467 shRNA1 (KD1) (5'-TTAGCTCATGAACGCAGACC-3') and shRNA2 (KD2) (5'-AGTCGTACCACAGAACTGTC-3') under the human H1 RNA polymerase III promoter has been described [20]. A si-Zfp467 expression unit was generated by inserting hybridized oligo DNAs into pBShH1 plasmid DNA. After sequence confirmation, two si-Zfp467 expression units were excised from the pBShH1 plasmid DNA by XbaI and XhoI digestion and inserted into XbaI/XhoI sites of the FG12 lentiviral vector. A scrambled si-RNA was used as a control.

### Virus production and titration

We generated lentiviral vector stocks using an HIV-1-based reporter virus, packaging plasmid pCMV R8.2ΔVpr and the VSV-G envelope protein-coding plasmid by calcium phosphate-mediated transient transfection of 293T cells. Culture supernatants were harvested on 2 days after transfection. Lentiviral vector particles were concentrated 300-fold by ultracentrifugation. Concentrated virus stocks at a multiplicity of infection of 10 were titrated on 293 T-cells based on EGFP expression.

### Transfection and transplantion of cells

ADSCs were transduced ex vivo with the VSV-G pseudotyped shRNA vector at a multiplicity of infection of once per day for 2 days with gentamicin. Transduction efficiency was analyzed by quantifying EGFP expression by flow cytometry 48 h after transduction.

### Detection of ADSCs after intravenous injection

After eight-week-old female ddY mice had been ovariectomized, ADSCs (2 × 10^6 ^cells/mouse) labeled with iron oxide (Ferridex, Berlex Laboratories Inc, Wayne, NJ) were injected into OVX mice via tail vein on postoperative day 4 and sacrificed at day 24 after injection. Bone tissues were fixed and decalcified with EDTA solution. After washing, paraffin-embedded specimens were sectioned, deparaffinized, incubated with 1% potassium ferrocyanide in 1% HCl for 30 min, and counterstained with nuclear fast red. Six animals were included in each group.

### Real-time quantitative, Western blotting and ELISA

For detection of mRNA level, total RNA was isolated with TRIzol (Invitrogen, Carlsbad, CA), according to the manufacturer's instructions. About 2 μg of total RNA was reverse transcribed using Moloney Murine Leukemia Virus Reverse Transcriptase (Promega, Madison, WI) with oligo dT at 42°C for 1 h. Detailed information on PCR, including primer sequences and cycles, is provided in Table 1.

**Table 1:** Nucleotide sequences of 10 primers for qRT-PCR.

Gene	Primer	Sequence (5' - 3')	Melting temperature (°C)	Product size (bp)
β-actin	Forward	5'-GTGGAGTGCCCAAGCACCA- 3'	52	213
	Reversed	5'-CTCTAATGTCACGACGATTTC-3'		
Adiponectin	Forward	5'-AGGAATTCTACTGCCGTCGA-3'	55	179
	Reversed	5'-TCAAGGCGAGCTCGTATTTGA-3		
AIP	Forward	5'-GCTAGCAAACCACCTAAGTA-3	54	207
	Reversed	5'-GTGGTCCAGAACATAGTAGA-3		
Zfp467	Forward	5'-CACGTGACACCTACCAAGTA-3	55	186
	Reversed	5'-CCGTAACAGGTATGAGTAGA-3		
OCN	Forward	5'-GCCCAGCTGTAACCACGATA-3	56	126
	Reversed	5'-CAGGTCCATAAGGTAGTAGA-3		
OPN	Forward	5'-GCATGCTACACCAACCAGTA-3	52	198
	Reversed	5'-CAGAAGTGGTGTACAGTAGA-3		
COL I	Forward	5'-GCAGCCCCATAGACGCCGTA-3	51	171
	Reversed	5'-CAGAGGTCATCCACCAATAGA-3		
BSP	Forward	5'-GCAGAGTAGTACTGCAGGTA-3	55	190
	Reversed	5'-CAGAGGTGTCCATAAGTAGA-3		
LPL	Forward	5'-GCGAGTGTAGCTCAGCCGTA-3	53	185
	Reversed	5'-CAGGTCCATAGAGGTAGTAGA-3		
PPARγ	Forward	5'-GTAGAGTGGCACAGTGGGTA-3'	52	313
	Reversed	5'-TTACTTGCCATCAAGATTGTC-3'		

For Western blotting, cells were lysed by addition of lysis buffer containing 20 mM Tris-HCl, pH 7.5, 150 mM NaCl, 1% NP-40, 0.5% Na-deoxycholate, 1 mM EDTA, 0.1% SDS, protease inhibitors (Complete tablets, Roche Molecular Biochemicals, Mannheim, Germany), 1 mM Na_3_VO_4_, and 1 mM NaF. Proteins were separated by 10% SDS-PAGE, transferred to a nitrocellulose membrane, and probed with Zfp467 antibody (R&D Systems Inc., Minneapolis).

Urine deoxypyridinoline was assayed by competitive enzyme immunoassay using the MicroVue DPD EIA kit (Quidel Corporation, Santa Clara, CA).

### Flow cytometry

ADSCs were harvested by trypsinization and centrifugation, washed twice with cold PBS, and fixed with cold 75% ethanol at 4°C overnight. The fixed cells were collected, washed twice with PBS and suspended in PBS. Flow Cytometry was using to array cultured ADSCs at day 7 with FITC-conjugated anti-CD29, FITC-conjugated anti-CD44, PE-conjugated anti-CD45 and PE-conjugated anti-HLA-DR antibody (Abcom Inc., Cambridge, Massachusetts), respectively.

### Gene expression profiling analysis using cDNA microarray

The cDNAs retrotranscribed from an equal amount of mRNA from control (ADSCs at day 0), adipocyte and osteoblast derived from ADSCs at day 14, were labeled with Cy3 and Cy5 fluorescence as probes, respectively. Fluorescence intensity was measured for each gene spot. After background subtraction and the whole-chip data normalization, the ratios of gene expression differences between these sets of samples were obtained. BioStar H-40s microarray consisted of 4096 novel or known genes including control system and effective genes (provided by Biostar Genechip Inc). The control system consists of 96 housekeeping genes as loading control; 16 plant genes, and spotting solution (without DNA, 16 spots) as negative control spots in the array. The chips were scanned with a ScanArray 4000. The acquired images were analyzed using GenePix Pro 3.0 software.

### Microcomputed tomography and histological analysis of bone

A total of 2 × 10^6 ^cells were injected into sham-operated or ovariectomized female ddY mice (8-week-old, Central lab animal, Korea) via tail vein on postoperative day 4 and sacrificed at day 24 after injection (n = 6 per group). Microcomputed tomography (μCT) and histological analysis were performed, as reported previously [7,21]. Trabecular morphometry within the proximal tibia was quantified using high resolution microcomputed tomography (μCT, Skyscan 1076 μCT, Aartselaar, Belgium). From μCT data, bone loss indices, including bone volume/total volume (BV/TV), trabecular number (Tb.N), and bone mineral density (BMD) were assessed. For analysis of bone formation, mice were injected with calcein (10 mg/kg) on postoperative day 14 and day 21 and were sacrificed at postoperative day 24.

OC number (no.) per bone surface (NOc/BS) and OB number per bone surface (NOb/BS) were analyzed by tartrate-resistant acid phosphatase (TRAP) staining on the 7-mm thick sagittal sections, as described previously [22]. TRAP positive cells containing one or more nuclei and sitting on the surface of the trabeculae were defined as osteoclasts. One section per animal was analyzed for these parameters.

### Statistical analysis

The results were expressed as mean ± SD. The data were treated by Student's t test to determine statistical significance. We used nonparametric tests (Mann-Whitney test), if appropriate, to compare differences. P < 0.05 was considered statistically significant. Statistical analysis was performed using SPSS 12.0 software.

## Results

### ADSC morphology and characteristics

**Figure 1:**
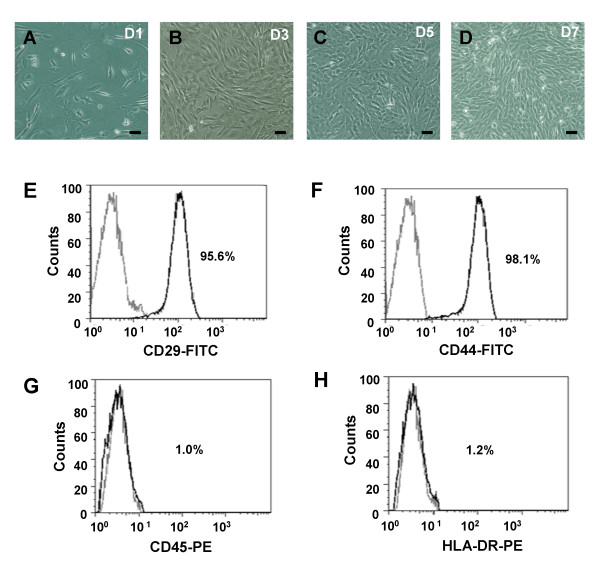
**Microphotographs and Flow Cytometry of cultured ADSCs**. (A-D) Microphotographs of cultured ADSCs at day 1, 3, 5 and 7, respectively. Bar scale = 20 μm. Flow Cytometry was using to array cultured ADSCs at day 7 with FITC-conjugated anti-CD29 (E), FITC-conjugated anti-CD44 (F), PE-conjugated anti-CD45 (G) and PE-conjugated anti-HLA-DR antibody (H), respectively.

**Figure 2:**
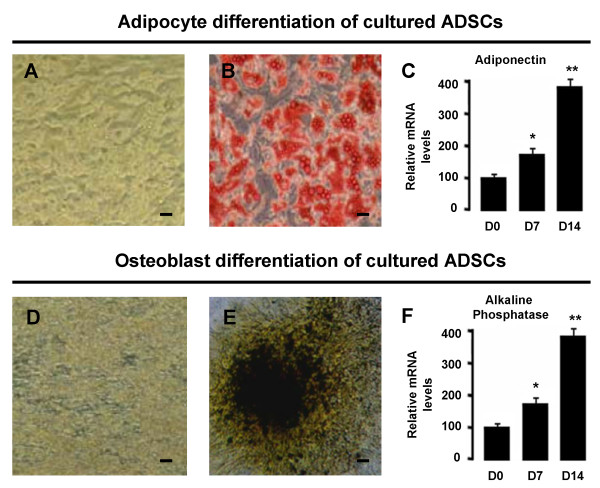
**Adipocyte and osteoblast differentiation of cultured ADSCs**. Microphotographs of ADSCs differentiated into adipocytes (A) and osteoblasts (D) at day 14. Bar scale = 50 μm. Differentiation in cultured ADSCs was monitored including oil red O staining of lipid droplets in terminal adipocyte differentiation (B) and von Kossa staining of calcium deposition in the extracellular matrix in terminal osteoblast differentiation (E). The qRT-PCR analysis of mRNA levels of specific adipogenic (adiponectin) (C) and osteogenic (alkaline phosphatase) markers (F) at day 7 and day 14 of differentiation as compared to day 0. The data are representative of three independent experiments.

ADSCs were obtained from the abdomens of 3- to 6-week-old mice and primary ADSCs displayed typical fibroblast-like morphology and were maintained for long-term culture in vitro (Figure 1A-D), and were capable of differentiating into adipocytes (Figure 2A-C) and osteogenic cells (Figure 2D-F) under specific induction [19]. Cell surface marker expression by ADSCs isolated from human adipose tissue was characterized. Flow cytometric analysis of ADSCs revealed that high levels of MSC-related CD29 and CD44 were expressed (Figure 1E, F); however, hematopoiesis-related antigens CD45 and immunogenicity markers HLA-DR were not expressed (Figure 1G, H).

In additional, the ability of ADSCs to differentiate into adipocyte and osteoblast lineages at passage 3 was experimentally confirmed by Oil red O staining (Figure 2B), von Kossa staining (Figure 2E) and the mRNA levels of specific adipogenic (adiponectin) (Figure 2C) and osteogenic (alkaline phosphatase) markers (Figure 2F), respectively. These results showed that ADSCs have multilineage potential and characteristics of MSCs.

### Gene expression profiling changes of adipocyte and osteoblast differentiation from ADSCs

**Figure 3:**
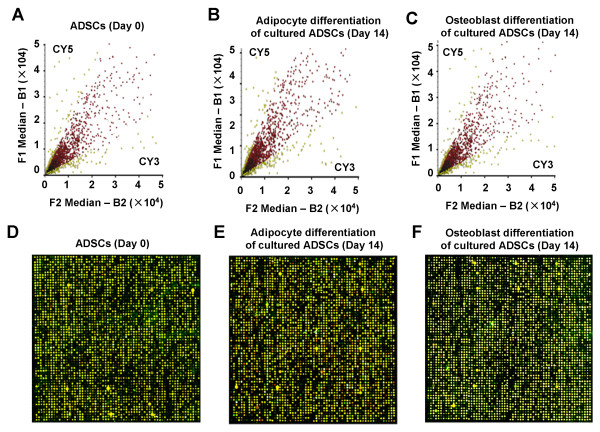
**Gene expression profiling of ADSCs**. The Gene expression profiling of adipocyte and osteoblast derived from ADSCs at day 14 and undifferentiated ADSCs (Day 0) were measured using cDNA microarray. The scatter plots that were plotted with Cy3 and Cy5 fluorescent signal values displayed a quite disperses pattern in distribution (A-C). Some yellow spots distributed beyond or far from 45°line indicated the existence of abnormal gene expressions in three groups (D-F).

Next, we studied the difference of gene expression profile changes between adipocyte and osteoblast differentiation from ADSCs at day 14 and to screen the critical genes in the early differentiation stage by cDNA microarray. The scatter plots that were plotted with Cy3 and Cy5 fluorescent signal values displayed a quite disperses pattern in distribution. Most of the spots gathered around a 45° line, in which red spots represented the area where the signal intensities varied between 0.5 to 2-fold compared with those of the control. Some yellow spots distributed beyond or far from 45° line indicated the existence of abnormal gene expressions between adipocyte and osteoblast derived from ADSCs at day 14. Their signal intensities were 2 times more than that of the control (Figure 3A-C). As shown in Figure 3D-F, 298 genes were screened out that exhibited different expressions between ADSCs, adipocyte and osteoblast derived from ADSCs, there were 175 up-regulated and 123 down-regulated genes in the gene expression profiles of ADSCs which was 2 times of that in adipocyte and osteoblast derived from ADSCs.

### Zfp467 knockdown by shRNA-expressing vectors

**Figure 4:**
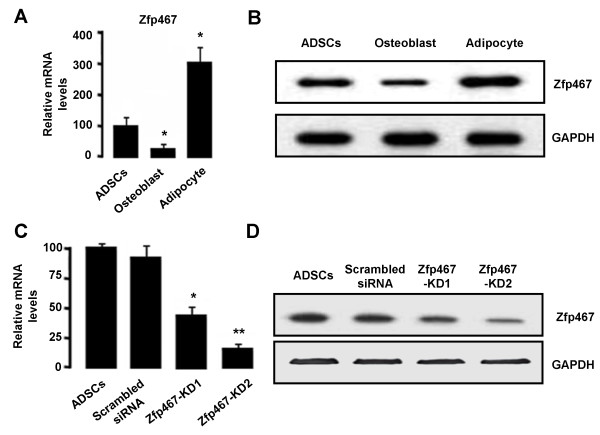
**The mRNA and protein levels of Zfp467 in cultured adipocyte, osteoblast and undifferentiated ADSCs**. The mRNA and protein levels of Zfp467 in adipocyte and osteoblast derived from ADSCs at day 14 and undifferentiated ADSCs (Day 0) were measured using qRT-PCR (A) and Western blot analysis (B). The data are representative of three independent experiments. The two si-Zfp467 vector (pSilencer4.1-si-Zfp467 with neomycin resistance), which produces specific siRNA (Zfp467-KD1 and -KD2) were used in the experiment. Scrambled siRNA was used as a control for siRNA-Zfp467. The efficiencies of Zfp467 RNAi in ADSCs were evaluated using qRT-PCR (C) and Western blot analysis (D).

We then choose Zfp467, a novel regulator of osteoblast and adipocyte commitment [18], to further study the molecule mechanism of ADSCs differentiation. The mRNA and protein levels of Zfp467 in adipocyte and osteoblast derived from ADSCs (Day 14) and control (ADSCs, Day 0) were measured using qRT-PCR and Western blot analysis, respectively. The results showed that the mRNA levels of Zfp467 in adipocyte derived from ADSCs was notably increased when compared with that in the control, however the mRNA levels of Zfp467 was significantly decrease after ADSCs differentiation to osteoblast (Figure 4A). The change of Zfp467 protein levels showed to be the same on the mRNA levels of Zfp467 in three groups (Figure 4B).

We then created a lentiviral vector encoding Zfp467 shRNA for long-term expressing Zfp467 siRNA to knockdown Zfp467 gene expression. We used two different siRNA sequences to target human Zfp467 gene, termed as Zfp467 shRNA-1 (KD1) and Zfp467 shRNA-2 (KD1). Control cells were transfected with lentiviral vector expressing scrambled siRNA. Zfp467 shRNA-1 and shRNA-2 dramatically reduced Zfp467 expression at both mRNA (Figure 4C) and protein levels (Figure 4D). The inhibition rate of Zfp467 shRNA-1 and Zfp467 shRNA-2 were 64.4% and 83.6%, respectively in ADSCs compared with the mock and control group at mRNA levels (Figure 4C). However, shRNA-2 caused more profound reduction of Zfp467 gene expression when compared to shRNA-1, reflecting a sequence-specific effect of the siRNA (Figure 4C, D).

### Effect of Zfp467 shRNA on adipocytes and osteoblasts differentiation

**Figure 5:**
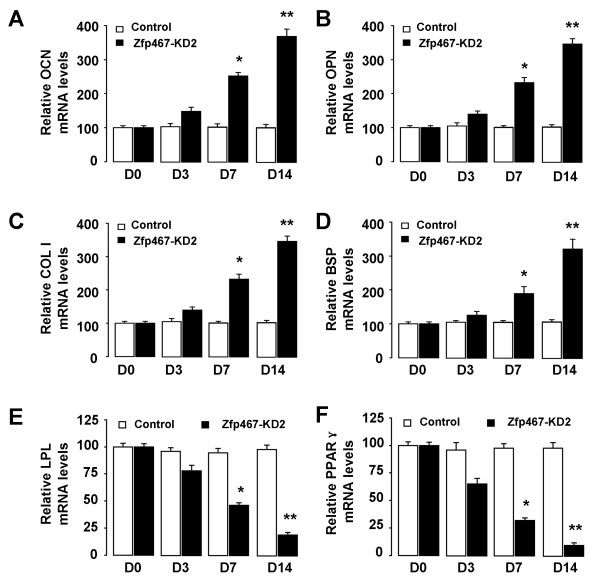
**The expressions of specific osteogenic and adipogenic genes**. The expressions of specific osteogenic and adipogenic genes in ADSCs were evaluated by real-time RT-PCR at 0, 3, 7 and 14 days after siRNA-Zfp467 transduction. Specific osteogenic marker Osteocalcin (OCN) (A), Osteopontin (OPN) (B), Collagen I (COL I) (C) and bone sialoprotein (BSP) (D) were up-regulated in si-Zfp467 transduced cells. Specific adipogenic marker lipoprotein lipase (LPL) and peroxisome proliferator-activated receptor gamma (PPARγ) were down-regulated in si-Zfp467 transduced cells (Mean ± SEM, n = 3). * p < 0.05 vs. control group; ** p < 0.01 vs. control group.

As mentioned above, in two representative subclones, Zfp467 shRNA-2 caused more profound reduction of Zfp467 gene expression when compared to shRNA-1. Hereafter, we mainly used the Zfp467 shRNA-2 subline for further analysis. The expressions of specific osteogenic and adipogenic genes were evaluated by real-time RT-PCR at 0, 3, 7 and 14 days post-transduction. Our results indicated that specific osteogenic marker Osteocalcin (OCN), Osteopontin (OPN), Collagen I (COL I) and bone sialoprotein (BSP) were up-regulated in si-Zfp467 transduced cells (Figure 5A-D). Specific adipogenic marker lipoprotein lipase (LPL) and peroxisome proliferator-activated receptor gamma (PPARγ) were down-regulated in si-Zfp467 transduced cells (Figure 5E-F). The statistical analysis showed that the plasmid Zfp467 shRNA could have significant inhibitive effects on the mRNA levels of specific adipogenic gene at day 7 and 14 when compared with that of adipogenic gene at day 0 and 3 (p < 0.05 and p < 0.01, respectively) (Figure 5A-D), however, RNA interfere of Zfp467 significantly enhanced the mRNA levels of specific osteogenic gene at day 7 and 14 when compared with that of osteogenic gene at day 0 and 3 (p < 0.05 and p < 0.01, respectively) (Figure 5E-F), which suggested Zfp467 involved in the ADSCs differentiated into adipocytes and osteoblasts.

### Effects of systemic transplantation of ADSCs on OVX-induced bone loss

**Figure 6:**
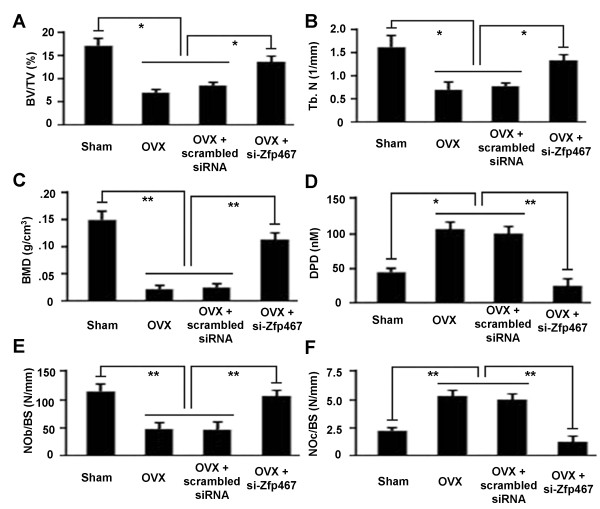
**Effects of systemic transplantation of ADSCs on OVX-induced osteoclasts formation and bone destruction**. Tibiae from sham-operated (sham) and OVX (OVX, OVX + Scrambled siRNA and OVX + si-Zfp467) mice were examined by μCT. 3D reconstruction of tibiae revealed that bone mass in OVX mice treated with si-Zfp467 ADSCs (OVX + si-Zfp467) significantly increased when compared with that in OVX or ADSCs (with scrambled siRNA) transplanted OVX mice. (A-C) Histograms represent the 3D trabecular structural parameters in tibiae: bone volume fraction (BV/TV), trabecular number (Tb.N), and bone mineral densities (BMD). (D) The levels of DPD in four groups were assayed by competitive enzyme immunoassay using the MicroVue DPD EIA kit. (E) OC number (no.) per bone surface (NOc/BS) was using to indicate quantification of OC cells. (F) OB number per bone surface (NOb/BS) was using to indicate quantification of OB cells. *P < 0.01, **P < 0.05. Data represent mean ± SD. n = 6.

We performed μCT analysis to determine the impact of ADSCs and Zfp467 on OVX-induced osteoporotic mice. Systemic transplantation of ADSCs with si-Zfp467 into OVX mice prevented OVX-induced bone loss in mice. When compared with OVX or ADSCs (with scrambled siRNA)-transplanted OVX mice, bone loss indices, including bone volume fraction, trabecular number, and bone mineral density in si-Zfp467-ADSCs transplanted OVX mice were restored to normal (the level of sham-operated mice) (Figure 6A-C).

The recovery effect of ADSCs with si-Zfp467 on bone loss was confirmed by measurement of the concentration of urinary deoxypyridinoline, a useful marker of bone resorption. Compared with OVX or ADSCs (with scrambled siRNA)-transplanted OVX mice, a significantly lower level of urinary deoxypyridinoline (DPD) was exhibited by si-Zfp467-ADSCs transplanted OVX mice (Figure 6D). Mineral apposition rate and bone formation rate, as measured by calcein label-based analysis, were restored to the level of sham-operated mice in si-Zfp467-ADSCs transplanted OVX mice. Of particular interest, histologic analysis showed that the numbers of osteoblasts adhering to trabecular bone surfaces were significantly increased in si-Zfp467-ADSCs transplanted OVX mice compared with OVX or ADSCs (with scrambled siRNA)-transplanted OVX mice (Figure 6E) (p < 0.01) and the numbers of TRAP-positive osteoclasts evidently decreased, which suggested that systemic transplantation of si-Zfp467-ADSCs in OVX mice might affect proliferation or differentiation of osteoblasts and osteoclasts. From these data, we confirmed that Zfp467 plays an important role in ADSCs to alleviate OVX-induced osteoporosis through regulating ADSCs differentiation to adipocyte and osteoblast.

## Discussion

In this study, we showed that ADSCs-based therapy via systemic transplantation could be effective in alleviating OVX-induced osteoporosis by a mechanism predominantly mediated through suppression of Zfp467.

Osteoporosis is a prevalent bone disease that is characterized by loss of bone mass and strength, leading to fragility fracture [23]. Osteoblasts, which are derived from mesenchymal stem cells, are ultimately responsible for bone formation; osteoclasts are derived from hematopoietic cells and are capable of resorbing bone. During adult life, bone is continuously remodeled by orchestrated cross-talk between osteoblasts and osteoclasts [24], and an imbalance in their function results in decreased bone quality, most commonly represented by the osteoporotic phenotype. A relatively higher bone resorption activity by osteoclasts than bone formation activity by osteoblasts leads to bone loss. High-turnover and low-turnover osteoporotic phenomena are known to be achieved by excessive bone resorption and reduced bone formation during bone remodeling, respectively [25].

Recent progress in stem cell biology has provided a promising strategy for treatment of osteoporosis. Therapeutic potential for bone regeneration by systemic transplantation of genetically manipulated MSCs coexpressing CXCR4 and Runx2 in glucocorticoid-induced osteoporotic mice has been recently suggested [26]. Lee K, et al. showed that hASC-based therapy via systemic transplantation could be effective in bone repair by a mechanism predominantly mediated through secretion of paracrine factors by hADSCs [27].

Considering the fact that the pathogenetic mechanisms underlying osteoporosis cover multiple sets of dynamic parameters, a systemic approach using stem cell transplantation is attractive for treatment of osteoporosis. Our in vivo results revealed that OVX-induced bone loss was restored by systemic transplantation of ADSCs into recipient OVX mice. We further showed that si-Zfp467-ADSCs-injected OVX mice exhibited an increase in the number of both osteoblasts and osteoclasts. This could be explained by the balance between bone resorption and bone formation. The levels of bone resorption by osteoclasts did not exceed those of bone formation by osteoblasts, resulting in a net increase of bone mass. These findings indicate that RNAi of Zfp467 in ADSCs can rescue estrogen deficiency-induced bone loss by simultaneous stimulation of osteoblast-mediated bone formation and osteoclast-mediated bone resorption in recipient OVX mice.

## Abbreviations

ADSCs: Adipose derived stem cells; ALP: Alkaline phosphatase; BSP: Bone sialoprotein; COL I: Collagen I; OCN: Osteocalcin; OPN: Osteopontin; OVX: Ovariectomized; PPARγ: Peroxisome proliferator-activated receptor gamma; qRT-PCR: Quantitative real-time reverse transcription polymerase chain reaction; Zfp: Zinc finger protein.

## Competing interests

The authors declare that they have no competing interests.

## Authors' contributions

LY, LP and LC carried out the most studies and drafted the manuscript. LC carried out the immunoassays. JYC, ZL and XZ participated in the design of the study and performed the statistical analysis. LY and DF conceived of the study, and participated in its design and coordination. All authors read and approved the final manuscript.
